# Nanoscale chemical and structural study of Co-based FEBID structures by STEM-EELS and HRTEM

**DOI:** 10.1186/1556-276X-6-592

**Published:** 2011-11-15

**Authors:** Rosa Córdoba, Rodrigo Fernández-Pacheco, Amalio Fernández-Pacheco, Alexandre Gloter, César Magén, Odile Stéphan, Manuel Ricardo Ibarra, José María De Teresa

**Affiliations:** 1 Laboratorio de Microscopías Avanzadas (LMA), Instituto de Nanociencia de Aragón (INA), Universidad de Zaragoza, Zaragoza, 50018, Spain; 2 Departamento de Física de la Materia Condensada, Universidad de Zaragoza, Facultad de Ciencias, Zaragoza, 50009, Spain; 3 STEM Group-Laboratoire de Physique des Solides (CNRS-UMR 8502), Université Paris-Sud, Bat. 510, Orsay Cedex, 91405, France; 4 Fundación ARAID, Zaragoza, 50004, Spain; 5 Instituto de Ciencia de Materiales de Aragón (ICMA), CSIC-Universidad de Zaragoza, Facultad de Ciencias, Zaragoza, 50009, Spain

**Keywords:** Co deposits, FEBID, EELS, HRTEM

## Abstract

Nanolithography techniques in a scanning electron microscope/focused ion beam are very attractive tools for a number of synthetic processes, including the fabrication of ferromagnetic nano-objects, with potential applications in magnetic storage or magnetic sensing. One of the most versatile techniques is the focused electron beam induced deposition, an efficient method for the production of magnetic structures highly resolved at the nanometric scale. In this work, this method has been applied to the controlled growth of magnetic nanostructures using Co_2_(CO)_8_. The chemical and structural properties of these deposits have been studied by electron energy loss spectroscopy and high-resolution transmission electron microscopy at the nanometric scale. The obtained results allow us to correlate the chemical and structural properties with the functionality of these magnetic nanostructures.

## Background

Despite its great potentiality for the synthesis of well-controlled metallic functional nanostructures for magnetotransport applications, the use of focused electron beam induced deposition [FEBID] [1,2] for such purpose has been quite limited, mainly due to the low purity of the deposits grown in this way. Organic precursors are usually dissociated as the source of metallic content, resulting in a mixture of carbon, metal, and oxidized material, thus producing inappropriate properties for the desired application in some cases. In the case of cobalt-based deposits, Co_2_(CO)_8 _is commonly used as the precursor gas, and the first experiments carried out only achieved a relatively low Co content [3,4].

As a consequence, different strategies have been tested to improve the cobalt content, including systematic studies of the influence of various deposition parameters [5-8] or the use of a heated substrate [9-11], which induces high precursor molecule decomposition and increases significantly the metallic content of these structures, implying a direct impact in their properties and their applications [12]. When high beam currents are used in the FEBID process, the cobalt content of the deposits can be higher than 90%, as measured by electron dispersive X-ray spectroscopy [EDS] [7]. It has been argued that beam-induced heating is one of the mechanisms responsible for the increase of metallic content with the electron current [6,7,11]. Beyond the confirmation of a much higher Co content in these types of FEBID deposits by EDS, no study had been performed at the nanoscale so far to clarify the nature and electronic state of cobalt inside the metallic deposit.

The aim of this paper is to analyze the valence state and crystal structure of Co in FEBID deposits so as to find an explanation from a chemical and structural point of view at the micro and nanoscale to the magnetic, chemical, and structural properties studied previously. For that, the analytical techniques developed and implemented in a (scanning) transmission electron microscope [(S)TEM] are the most appropriate tools for this kind of local observation. For this purpose, electron energy loss spectroscopy [EELS] is the ideal technique for analyzing the oxidation state and the chemical environment at the local scale of the three elements present in the deposits: carbon, oxygen, and cobalt. In a STEM, EELS spectra can be highly resolved spatially and correlated to their position in the sample by the simultaneous acquisition of high-angle annular dark field [HAADF] images. On the other hand, the analysis of high-resolution transmission electron microscopy [HRTEM] images yields the information on the crystalline structure at an atomic scale. Both techniques confirm the high metallic content of the grown deposits when a high electron beam current was used.

## Methods

In order to study the influence of a deposition parameter such as the electron beam current [*I*_e_] in the microstructure and composition of the Co-based FEBID nanodeposits at the nanometer scale, two FEBID magnetic nanodeposits were fabricated at room temperature using a field emission gun scanning electron microscope electron column. The deposits were grown on an oxidized silicon wafer SiO_2_//Si substrate using a working voltage of 30 kV. In order to compare the effect of the working current *I*_e _on the final metallic content, one of the deposits was grown at a low *I*_e _(in picoampere range) and another one at a high *I*_e _(in nanoampere range). In both cases, the Co_2_(CO)_8 _precursor gas was brought onto the substrate surface by means of a gas injection system and decomposed under the focused electron beam. Common parameters for this rectangular shape Co-based deposition process were the following: Co nanostructures with dimensions (width × length × thickness) = 0.5 × 1.0 × 0.2 μm^3^; Vol/dose = 5 × 10^-4 ^μm^3^/nC; dwell time = 1 μs; beam overlap = 50%; refresh time = 0 s; base chamber pressure = 1 × 10^-6 ^mbar; process chamber pressure = 4.3 × 10^-6 ^mbar; scan strategy = bottom to top in serpentine mode; vertical distance between gas injection system needle and substrate = 135 μm; horizontal distance = 50 μm; and pitch = 2.21 nm (deposit 1, 0.044 nA), 13.16 nm (deposit 2, 2.4 nA).

Following the nanodeposit growth, *in situ *EDS analysis has been performed on them (deposit 1, Co:C:O 64:17:19; deposit 2, Co:C:O 93:5:2). Prior to the lamella preparation, the Co deposits were covered with a layer of FEBID-grown platinum and a second layer of focused ion beam induced deposition [FIBID]-grown platinum. This standard procedure was carried out to protect the deposit from the ion beam damage during lamellae preparation. The *in situ *lift-out and cross section TEM lamellas of the Co deposits have been fabricated using the focused ion beam present in the same equipment. The final thinning and polishing have been done at an ion beam acceleration voltage of 5 kV to decrease the amorphization layer. The final lamella thickness was lower than 50 nm.

The microstructure of the nanodeposits has been investigated by HRTEM, whose results were obtained using an image Cs-aberration-corrected FEI Titan Cubed at 300 kV (FEI Company, Hillsboro, OR, USA). The correction of the spherical aberration of the objective lens leads to a spatial resolution of at least 0.1 nm.

The composition of the nanodeposits at the nanometer scale has been investigated by means of spatially resolved chemical analysis, carried out in a STEM VG HB 501 with a field emission gun operated at 100 kV and fitted with a Gatan 666 spectrometer (Gatan Inc., Pleasanton, CA, USA), optically coupled to a CCD camera. Spatially resolved EELS analysis was used to investigate the metallic cobalt content and the oxidation state in each deposit. Thus, the electron beam is scanned on the sample, and a series of spectra is collected for each point; thus, the obtained spectra can be compared as a function of the point of collection in the sample. This technique is known as spectrum-line or line scan acquisition [13]. For each line scan, spectra were acquired at steps of 1 nm, and then summed every five spectra for the calculation of intensity ratios of the Co L_2,3 _edge (*I*(L_2_) and *I*(L_3_), respectively). *I*(L_2_) and *I*(L_3_) were calculated as the intensity maximum for each edge. For the analysis of chemical composition as a function of growth direction, 200 spectra were acquired for each point, realigned, and summed. Principal components analysis [PCA] was applied to each series of spectra to decrease experimental noise and so as to obtain a better signal to noise ratio [14]. After applying PCA to each spectrum for a single point, five resulting consecutive spectra of a line scan were summed, and the intensities of the white lines were calculated after a power-law removal of the background and a linear fit below the lines. Therefore, the chemical state of Co has been first estimated by means of the intensity ratio of the L_2 _and L_3 _peaks. The reference values of *I*(L_2_)/*I*(L_3_), 0.31 for metallic cobalt and 0.27 for cobalt oxide [CoO] [15], were calculated using the same technique.

On the other hand, the relative O/Co concentrations were also calculated, integrating their respective signal intensities from a series of 200 summed EELS spectra at a single point inside the deposit and dividing by their respective cross sections. An energy dispersion of 0.2 eV/channel was used for the analysis of the fine structure for each element, whereas an energy dispersion of 0.5 eV/channel was used for the quantification of the relative amounts of each element, with a collection angle of 24 mrad and a convergence angle of 7.5 mrad. Both types of experiments had an acquisition time of 0.8 s/spectrum.

## Results and discussion

For each metallic deposit, a thorough chemical and structural analysis at the nanoscale has been performed by means of EELS and HRTEM. Together with the chemical analysis of the inner part of each deposit, spatially resolved analysis of the interfaces Pt-Co and SiO_2_-Co has also been performed to understand the differences in chemical composition between the core and the surface.

### Deposit 1: deposition parameters: *V*_e _= 30 kV, *I*_e _= 0.044 nA

**Figure 1:**
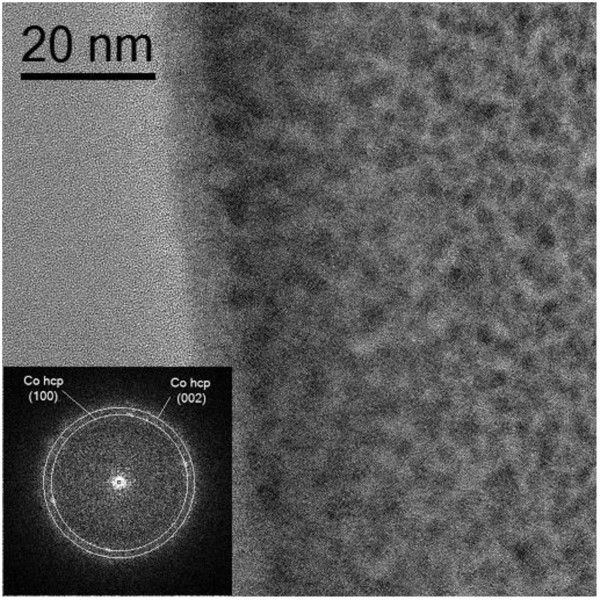
**HRTEM image and FFT (inset) of deposit 1**.

Direct observation of the HRTEM images (Figure 1) shows that the inside of the deposit is made of polycrystalline cobalt nanoparticles embedded in an amorphous carbon matrix, with approximately 2 to 3 nm of nanocrystal size. The presence of such small nanoparticles had been previously reported in the literature [6]. The HRTEM image is dominated by the amorphous contrast of the matrix, which gives rise to a fast Fourier transform [FFT] blurred by diffuse scattering. Only weak reflections associated to metallic hcp Co can be identified.

**Figure 2:**
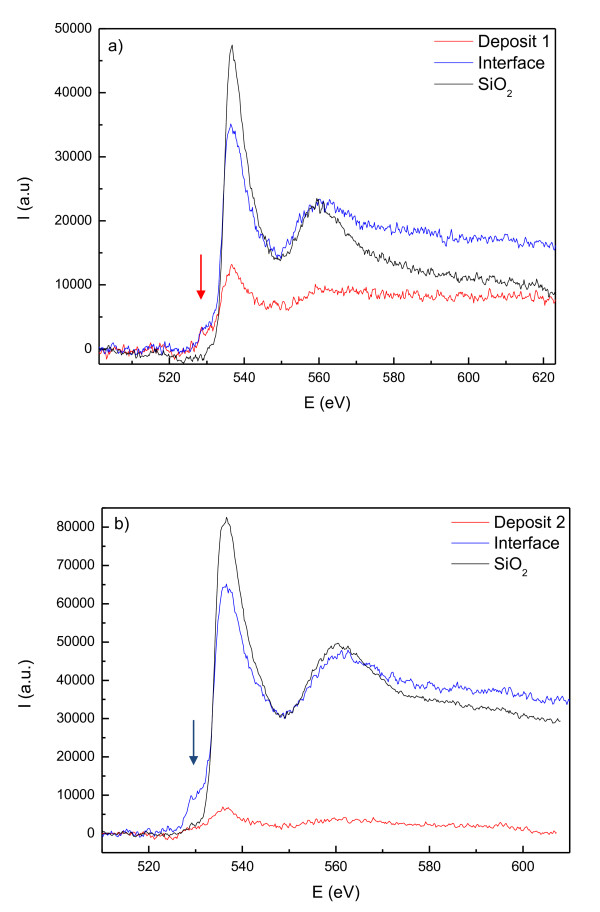
**O K edge (532 eV) spectra collected through the SiO_2_/Co interface**. The spectra were collected for deposits 1 (**a**) and 2 (**b**). As the probe scans through the SiO_2 _substrate, the interface between both materials, and finally the inner part of the deposit, the O K edge changes its shape (apparition of a small pre-peak, pointed with an arrow), practically disappearing at the inside of the microstructure for deposit 2.

**Figure 3:**
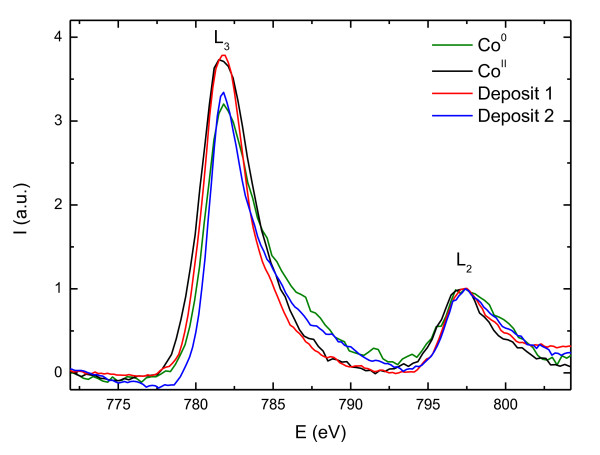
**Comparison of the EELS spectra**. Comparison of the EELS spectra of the Co L_2,3 _edge (at an energy of 779 eV) for deposits 1 and 2, and references for metallic cobalt and cobalt (II).

Though precise quantitative analysis of these kinds of granular samples is not feasible, the presence of metallic cobalt and cobalt oxide species is evident from the *in situ *compositional EDS analysis, where a 19% O content is observed. On the other hand, to understand the oxidation state of Co, the study of the L_2,3 _edge of cobalt and the K edge of oxygen in the EELS spectra can be very useful. The obtained spectra can be compared to EELS data in bibliography to check a shift in energy or any variation in the shape of the edges. Figure 2a shows the O K edge of deposit 1 collected at different points of the sample. Firstly, we confirm the existence of oxygen already in the spectrum collected at the core of the deposit, as observed by EDS. Furthermore, the presence of a small pre-peak at 531 eV at the O K edge fine structure of the deposit and the interface (not observed in the SiO_2 _spectrum) is a distinctive sign of the presence of CoO [16]. Also, the analysis of the energy loss near edge structure [ELNES] of the Co L_2,3 _edge can yield very useful information. Thus, the L_2_/L_3 _intensity ratio between the peaks of the white lines of the cobalt spectrum gives us an indication of the oxidation state of Co: when L_2_/L_3 _decreases, the oxidation state increases [17]. Figure 3 is a comparison of the white lines of Co L_2,3 _edge for deposits 1 and 2, and references of metallic cobalt and CoO. The EELS analysis for the first deposit shows the presence of oxidized cobalt, as it can be inferred from the shape of the L_2,3 _edge of the cobalt spectrum, and the low average L_2_/L_3 _ratio of around 0.27.

### Deposit 2: deposition parameters: *V*_e_= 30 kV, *I*_e_= 2.40 nA

**Figure 4:**
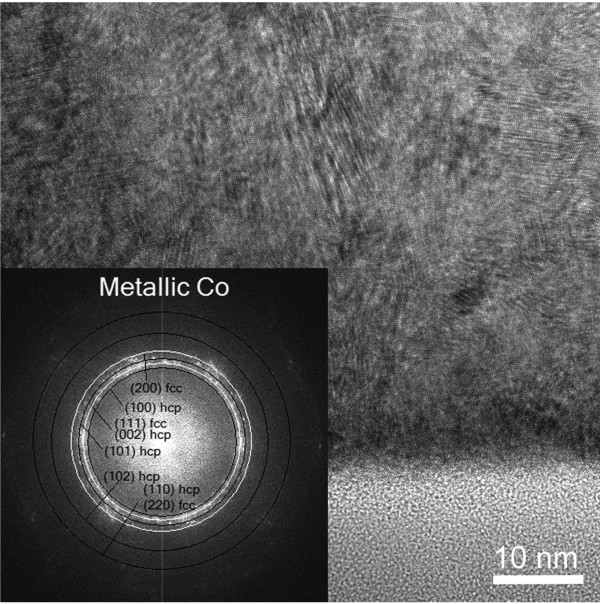
**HRTEM image and FFT (inset) of deposit 2**.

This sample shows a different microstructure and composition. The HRTEM image shown in Figure 4 presents a deposit made of cobalt nanocrystals with 7 to 10 nm in size. Cobalt grains are more regularly distributed and compact than in deposit 1. The microcrystalline structure obtained from the indexation of the digital diffractogram is compatible with a mixture of Co hexagonal closed-pack [hcp] and face-centered cubic [fcc] (inset in Figure 4). Regarding the EELS spectra, the ELNES study of the cobalt L_2,3 _edge yielded homogeneous, regular spectra with the characteristic white lines of metallic cobalt (Figure 3). Indeed for metallic Co, the L_3 _line shows a broad asymmetric shape compared to the narrower L_3 _line of Co oxide. The metallic character is confirmed by the L_2_/L_3 _ratio of 0.30 and negligible oxygen content (O/Co atomic ratio of about 0.04).

On the other hand, Figure 2b shows the EELS spectra at the O K edge region at the SiO_2_/Co interface. Looking into the fine structure at the interface between the SiO_2 _substrate and the cobalt structure, for the first nanometers of the growth of the deposit, one can observe the presence of a pre-peak at 531 eV, which is characteristic of the presence of CoO. As the probe scans the inner part of the deposit, the oxygen signal practically disappears. The presence of the CoO could be due to the existence of water molecules adsorbed on the substrate before the start of the FEBID process.

Table 1 is a summary of the preparation conditions for both samples and the quantitative ratio between oxygen and cobalt inside the deposit. The analysis of the ELNES yields information about the shape and the intensity of the major features both for Co L_2,3 _and O K edges. In order to estimate the oxidation state of cobalt, the intensity ratio between the peaks L_2_/L_3 _of the Co L_2,3 _edges was analyzed. As expected from previous EDS analyses, the deposit grown at a high beam current presents a lower O/Co ratio and a higher L_2_/L_3 _intensities ratio (close to that of metallic cobalt) than that grown at a low beam current. Therefore, EELS analysis shows that deposit 2 presented features characteristic of metallic cobalt, a fact confirmed by the absence of the O K edge for this particular deposit. On the other hand, oxidized cobalt was found in deposit 1, as it can be inferred from the shape of the L_2,3 _edge of the cobalt spectrum and the high L_2_/L_3 _ratio, as well as from the presence of a characteristic pre-peak at 531 eV for the O K edge feature.

**Table 1:** The preparation conditions for the samples and quantitative ratio between oxygen and cobalt

Deposit	*V*_e _(kV)	*I*_e _(nA)	O/Co	*I*(L_2_)/*I*(L_3_)
1	30	0.044	0.85	0.27
2	30	2.400	0.04	0.30

Summary of growth parameters, beam energy (*V*_e_) and current (*I*_e_), EELS quantification ratio between oxygen and cobalt and the average L_2_/L_3 _intensity ratios in Co L_2,3 _edge (see text for details).

However, for deposit 1 HRTEM images revealed the presence of Co hcp, a fact confirmed by the EELS analysis, which showed minor features of metallic cobalt. To understand the presence of CoO together with metallic Co in samples grown at a low beam current, we can assume that the particles that build up the deposit are so small that most of their atoms are present on the surface, oxidizing very easily and in a large proportion.

**Figure 5:**
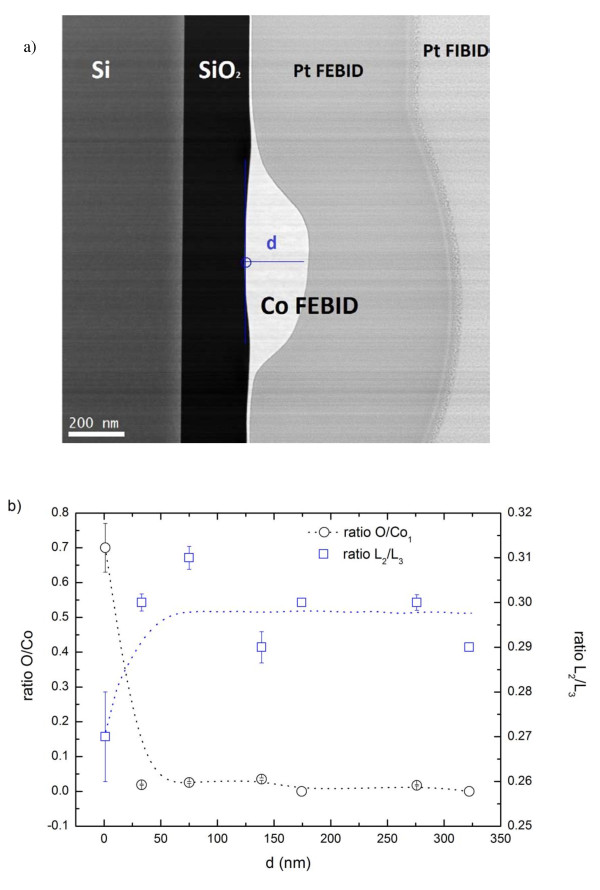
**Reference image and profiles of relative concentration**. **(a**) STEM-HAADF reference image of deposit 2. (**b**) Profiles of relative concentration of the O/Co and L_2_/L_3 _intensity ratios along the growth direction (blue line).

The homogeneity in composition and metallicity along the direction of deposition has also been studied for deposit 2, and it is illustrated in Figure 5. A relative quantification of the elements has been performed as a function of the growth direction of the deposit, confirming the metallic state of cobalt. The ratio O/Co is very low, lower than 0.1 all along the deposit. Only the first nanometers of deposition seem to be partially oxidized. This is in good agreement with the plotting of the L_2_/L_3 _intensity ratios along the deposit, which shows metallic ratios all through the deposit except in the early stages of growth where the intensity ratio falls down to 0.27 (Figure 5b).

Summarizing, in the growth conditions chosen, which are the same as those used in our previous publications [7,18,19], electron beam current plays a key role in the purity of the metallic content, thus being one of the driving force to produce cobalt in metallic state. The deposits grown at a high beam current have high cobalt content, whereas those grown at low beam currents have low cobalt content, where a significant amount of oxidized cobalt together with metallic cobalt has been detected. However, the FEBID process involves complex phenomena, and other relevant mechanisms have been also highlighted in literature using different deposition parameters. For example, the influence of autocatalysis [20] and the influence of the dwell time in the final composition [8] have been put forward. Thus, given a certain cobalt structure geometry, the final cobalt content will be determined by the set of the growth parameters (precursor flux, dwell time, refresh time, beam current) and not only by the beam current.

The strong differences in the microstructure and chemical nature of the deposits found in this systematic study might explain the different transport and magnetic properties reported in the literature for these Co-based nanostructures grown by FEBID. Thus, in the same deposition conditions chosen in the literature [7,18,19], samples grown at a high beam current show metallic electrical transport and ferromagnetic behavior [18,19] in sharp contrast with the semiconducting behavior exhibited by deposits grown at a low beam current [7].

## Conclusions

A thorough HRTEM and STEM-EELS study has been performed to investigate the microstructure of Co-based FEBID nanostructures grown using the organometallic precursor Co_2_(CO)_8_. In the same deposition conditions chosen in the literature [7,18,18], deposits grown at a high electron-beam current are formed by large cobalt nanocrystals, present more than 96% of metallic cobalt content, and exhibit metallic resistivity and ferromagnetic properties. Conversely, deposits grown at a low electron beam current present small isolated cobalt nanocrystals (5 to 7 nm in size) embedded in an amorphous carbon matrix with less than 80% of metallic cobalt content and semiconducting resistivity. In all cases, the high metallic content of these deposits produces fascinating magnetic properties, making them strong candidates in magnetic storage or magnetic sensing applications.

## Competing interests

The authors declare that they have no competing interests.

## Authors' contributions

JMDT and OS conceived the collaborative study and coordinated it. RC, AFP, JMDT, and MRI defined the geometry and the composition of the deposits. RC grew the deposits and carried out the TEM lamella preparation. RFP, AG, and OS performed the STEM and EELS characterization. CM and RC carried out the HRTEM characterization. All the authors discussed the results, contributed to the manuscript, and approved its final version.
